# Traditional Chinese Medicine and Sarcopenia: A Systematic Review

**DOI:** 10.3389/fnagi.2022.872233

**Published:** 2022-05-13

**Authors:** Chao-yang Guo, Yun-jing Ma, Shu-ting Liu, Ran-ran Zhu, Xiao-ting Xu, Zhen-rui Li, Lei Fang

**Affiliations:** ^1^Yueyang Hospital of Integrated Traditional Chinese and Western Medicine, Shanghai University of Traditional Chinese Medicine, Shanghai, China; ^2^School of Rehabilitation Science, Shanghai University of Traditional Chinese Medicine, Shanghai, China; ^3^Department of Rehabilitation, Shanghai East Hospital, Shanghai, China; ^4^Institute of TCM International Standardization, Shuguang Hospital Affiliated to Shanghai University of Traditional Chinese Medicine, Shanghai, China

**Keywords:** sarcopenia, traditional Chinese medicine, Chinese herb, Qigong exercise, acupuncture, systematic review

## Abstract

Sarcopenia has become a key challenge for healthy aging in older adults. However, it remains unclear whether traditional Chinese medicine can effectively treat sarcopenia. This systematic review analyzes the current evidence for the effect of traditional Chinese medicine (TCM) on sarcopenia. We searched for articles regarding sarcopenia treated by TCM in Cochrane library, PubMed, SinoMed, Web of Science, Embase, and the China National Knowledge Infrastructure (from inception until 10 December 2021). Two researchers independently screened the literature in accordance with the inclusion and exclusion criteria designed by PICOS principles. The risk of bias was assessed by the Cochrane Risk of Bias (ROB) tool. The quality of evidence was assessed by the grading of recommendations, assessment, development, and evaluation (GRADE). Participants’ characteristics, interventions, and the relevant results of the included studies were extracted and synthesized in a narrative way. The total number of participants in the 21 included studies was 1,330. Most of the studies evaluated physical function (*n* = 20) and muscle strength (*n* = 18), and a small number of studies (*n* = 6) assessed muscle mass. Overall, it was found that TCM had a positive impact on muscle strength (grip strength, chair stand test) and physical function (6-m walking speed, timed up and go test, sit and reach) in patients with sarcopenia, inconsistent evidence of effects on muscle mass. However, the small sample size of the included studies led to imprecision in the results, and the presence of blinding of the studies, allocation concealment, and unreasonable problems with the control group design made the results low grade. Among these results, the quality of evidence for grip strength (*n* = 10) was of medium grade, and the quality of evidence related to the remaining indicators was of low grade. This systematic review showed that traditional Chinese Qigong exercises and Chinese herbal medicine have a positive and important effect on physical performance and muscle strength in older adults with sarcopenia. Future high-quality multicenter randomized controlled trials (RCTs) with large samples are needed to determinate whether acupuncture and other therapies are effective in treating sarcopenia.

## Introduction

Sarcopenia is a progressive and pervasive age-related primary skeletal muscle disorder involving the accelerated loss of muscle strength and mass, which is associated with increased adverse outcomes, including fall fracture, disability, and mortality (Cruz-Jentoft and Sayer, 2019; Chen et al., 2020). Sarcopenia is recognized as an independent condition and was given an International Classification of Diseases-10 code in 2016. The 2019 European Working Group on Sarcopenia in Older People (EWGSOP) updated definition suggests that physical performance should be considered a measure of the severity of sarcopenia (Cruz-Jentoft et al., 2019). More than 50 million people worldwide currently have sarcopenia, and it is expected that more than 200 million people will have sarcopenia by 2050 (Sousa et al., 2016). Physical dysfunction is the primary problem caused by sarcopenia. In Western society, as many as 42% of individuals under 60 years of age have difficulties performing the activities of daily life (e.g., walking speed or standing up from a chair), 15–30% report being unable to lift or carry 10 pounds or more, and more than 30% are confronted with physical disabilities (Louie and Ward, 2010). Sarcopenia has become a key challenge for healthy aging in older adults because the early symptoms are not obvious and are difficult to prevent and manage effectively. The main international treatments for sarcopenia are currently exercise, high-protein nutritional supplementation, and medication (Cruz-Jentoft and Sayer, 2019). However, medications (such as sex hormones, growth hormone, vitamin D, testosterone, and angiotensin-converting enzyme inhibitors) and nutritional supplementation are ineffective (Li et al., 2022). Relevant evidence-based clinical practice guidelines were published in 2018 with strong recommendations for exercise as the primary treatment for sarcopenia (Dent et al., 2018). Exercise has a positive impact on the health of older adults, but aging is usually accompanied by a significant decline in the body’s motor organs and functions, thus emphasizing the importance of appropriate exercise intensity and exercise forms for older adults. Traditional Chinese medicine (TCM), the oldest existing medical model for maintaining health and curing diseases, includes intervention methods such as herbal medicine, acupuncture, moxibustion, Tuina, and Qigong (Chan, 2005). Qigong is a low-load aerobic exercise that is effective in preventing skeletal muscle atrophy with long-term practice (Penn et al., 2019). Chinese herbs, which are pure in nature, have few toxic side effects, and are effective in treating chronic diseases, may become an important treatment to sarcopenia. TCM has gradually been recognized by the public as a supplement to Western medical treatment (Yu et al., 2006). However, no previous study has evaluated the quality of the evidence for TCM for the treatment of sarcopenia.

Moreover, the mechanism by which TCM affects sarcopenia is not yet clear, and it may be related to enhancing neural recruitment, maintaining protein homeostasis, and reducing autophagy and inflammation. Therefore, we performed a systematic review to investigate the clinical evidence of current TCM therapies to treat sarcopenia and analyze their effects.

## Methods

### Search Strategy

Six electronic databases—Cochrane library, PubMed, SinoMed, Embase, Web of Science, and the China National Knowledge Infrastructure—(were screened from inception until 10 December 2021. The following Medical Subject Headings (MeSH) terms and their synonyms were used either singularly or in combination: (“Traditional Chinese exercise” OR “Qigong” OR “Tai Chi” OR “Gongfu” OR “Yi Jin Jing” OR “Ba Duan Jin”) AND (“Sarcopenia” OR “Sarcopenias”); (“acupuncture” OR “electric acupuncture”) AND (“Sarcopenia” OR “Sarcopenias”); (“Chinese herbal medicine” OR “herbal medicine” OR “herbs”) AND (“Sarcopenia” OR “Sarcopenias”). Reference lists of related reviews were searched for additional studies.

### Inclusion and Exclusion Criteria

The PICOS strategy was defined as follows: “P” (patient)—patients with sarcopenia of any age, gender, or race; “I” (intervention)—TCM; “C” (comparison)—comparison with a blank group or a different intervention group; “O” (outcome)—relevant indicators to evaluate muscle and body function; and “S” (study design)—randomized controlled studies, cohort studies, observational studies, or case–control studies.

Articles were included if they met all of the following criteria: 1. participants were diagnosed with sarcopenia based on any established definition (by a working group, a certain article, or clinical experience); 2. patients’ age ≥ 60 years; 3. the study included at least one traditional Chinese medicine treatment method, which could be herbal medicine, traditional Chinese exercise, acupuncture, and their combination. 4. Outcomes included muscle mass, muscle strength, physical function, or related biochemical indicators.

The exclusion criteria were as follows: 1. no original data were included (e.g., review, protocol, and abstract); 2. the participants had other accompanying diseases (e.g., cancer, liver cirrhosis, diabetes, stroke, depressive disorder, and metabolic syndrome).

### Data Extraction

Two researchers screened the literature independently in accordance with the inclusion and exclusion criteria and used the data extraction table to extract information, such as 1. basic information (e.g., author, year of publication, number of participants and age range); 2. group design measures and measured time points; 3. main outcome results. Any disagreement between the two authors was resolved by a consensus procedure. A third author was further consulted if the disagreement persisted.

### Risk of Bias and Quality Assessment of the Individual Studies

Two authors independently assessed the methodological quality of these studies using the Cochrane Risk of Bias (ROB) tool. The tool assessed the following seven characteristics: random sequence generation; allocation concealment; blinding of participants and personnel; blinding of outcome assessment; incomplete outcome data; selective reporting; and other bias. Based on the results of the risk of bias assessment of the included studies, the two authors independently assessed the quality level of the study results using GRADE software. According to the GRADE working group instructions, five factors, namely bias, inconsistency, indirectness, imprecision, and publication bias, lead to lower quality of evidence; and for each study, the quality of evidence was categorized into four levels: high, medium, low, and very low (Guyatt et al., 2008).

### Data Analysis

There was considerable heterogeneity in the included studies in terms of diagnosis of disease, sample size, outcome indicators, and interventions. We concluded that based on these heterogeneities and differences in study design, meta-analyses would not provide valid results and the risk of meta-analysis providing incorrect results was considerable. Participants’ characteristics, interventions, and the relevant results of the included studies were extracted and synthesized in a narrative way.

## Results

### Literature Search and Study Selection

**FIGURE 1:**
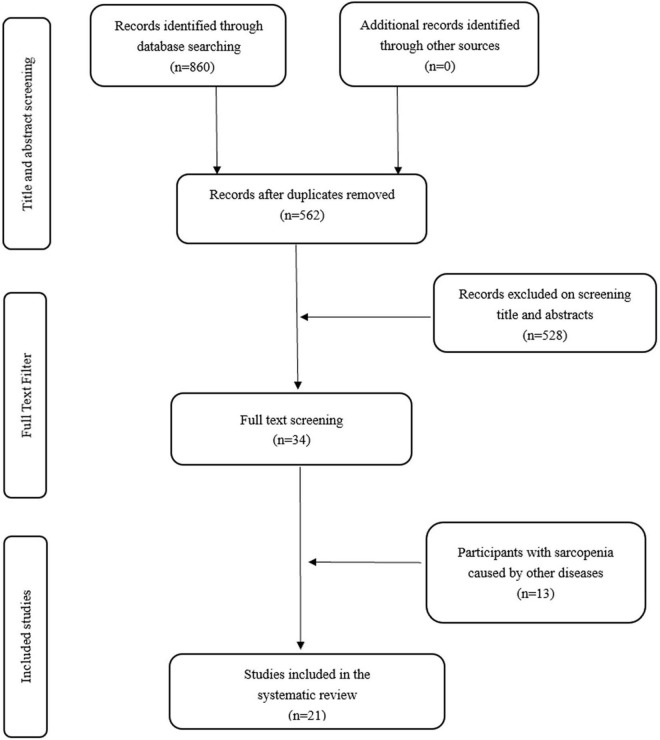
Flow diagram of the selection criteria for the study.

The selection process for this systematic review is shown in Figure 1. We used a 2-week period to identify 860 studies from the electronic database by the search strategy described above. After removing duplicate literature, 562 studies remained. After reading the titles and abstracts, 528 studies were excluded. The remaining 34 papers were read in full text. Full-text review removed 13 of these documents, leaving 21 studies that met all of the inclusion and exclusion criteria.

### Characteristics and Quality of Studies

The results of data extraction are shown in Tables 1, 2. A total of 19 trials were from China, one trial was from Brazil, and one trial was from Poland. A total of 21 studies involved a total of 1,330 participants, ranging from 15 to 214 per study. The age range of the included population was between 60 and 101 years. These studies included samples with different diagnostic criteria. Namely, nine studies referred to the Asia working group for sarcopenia (Ren et al., 2016; Zhao et al., 2016; Zhu et al., 2016, 2018; Wen et al., 2018; Liang et al., 2019; Chen, 2020; Zhou et al., 2020; Chen et al., 2021); three studies used European working group for sarcopenia diagnostic criteria (Soares Mendes Damasceno et al., 2019; Fang et al., 2020; Morawin et al., 2021); six studies (Yan et al., 2007; Gong et al., 2011; Jing et al., 2011; Liu et al., 2016; Wang et al., 2016; Zhu et al., 2017) complied with Roubenoff’s view of sarcopenia (Rosenberg, 2011); two studies did not provide diagnostic criteria, but only described subjects as having been diagnosed with sarcopenia in the hospital (Yang and Chen, 2016; Liang et al., 2019); and one study used the 2011 ISCCWG (International Sarcopenia Consensus Conference Working Group) diagnostic criteria (Liu et al., 2020). There was a large difference in outcome indicators in the included studies, and there were many scales and no specific content and scoring criteria. Twenty-one studies were divided into three broad categories by intervention methods: six herbal medicine studies, two acupuncture studies, and 13 Traditional Chinese exercise studies, with interventions ranging in duration from 10 days to 10 months.

**TABLE 1:** Characteristics of the included studies of Traditional Chinese Qigong exercises.

Study	Age	Number		Diagnostic criteria	Intervention		Outcome measures
		A	B		A	B	
Wang et al., 2016	66.79 ± 4.76	38	37	Roubenoff’s view	Yi Jing Jin, three times a week for 1 h each time for 12 weeks	No training	In-chair sitting-to-standing and squats, shoulder flexibility, sit and reach
Zhu et al., 2017	65.6 ± 11.4	32	31	Roubenoff’s view	Yi Jing Jin, once per day for 40 min for 12 weeks	Health education	Grip strength, in-chair sitting-to-standing and squats
Jing et al., 2011	68.22 ± 4.09	15	16	Roubenoff’s view	Yi Jing Jin, three times a week for 1 h each time for 8 weeks	No training	6-m gait speed, in-chair sitting-to-standing and squats, shoulder flexibility, sit and reach
Zhao et al., 2016	67.8 ± 3.8	6	6	AWGS	Yi Jing Jin combined with Tuina, three times a week for 40 min each time for 8 weeks	No training	Grip strength, 6-m gait speed
Liu et al., 2016	67.86 ± 6.86	31	30	Roubenoff’s view	Yi Jing Jin, three times a week for 8 weeks	No training	PT, AP, TW
Yang and Chen, 2016	77.5 ± 4.3	30	30	Unspecified	Yi Jing Jin, three times a week for 16 weeks	Tuina, three times a week for 16 weeks	Walking steps, lower extremity muscle strength score, skeletal muscle mass index
Fang et al., 2020	82.8 ± 8.5	15	14	EWGSOP	Yi Jing Jin, three times a week for 6 months	Health education	TUGT, MFES
Yan et al., 2007	>60	12	0	Roubenoff’s view	Yi Jing Jin combined with Tuina for 8 weeks	–	In-chair sitting-to-standing and squats
Zhu et al., 2018	67.17 ± 10.72	20	20	AWGS	Yi Jing Jin, once daily for 12 weeks	Tuina, three times a week for 12 weeks	FGA
Gong et al., 2011	66.4 ± 5.47	30	30	Roubenoff’s view	Yi Jing Jin, three times a week for 8 weeks	No training	PT, AP, TW
Zhu et al., 2018	88.8 ± 3.7	24	27	AWGS	Tai-Chi, five times a week for 40 min each time for 8 weeks	No training	Grip strength, 6-m gait speed, TUGT, FTSST
Morawin et al., 2021	73.6 ± 7.9	40	40	EWGSOP	Tai-Chi, for 10 months	Health education	6-m gait speed, Grip strength, CRP, TNFα
Zhou et al., 2020	72.67 ± 9.56	20	20	AWGS	BDJ, five times a week for 8 weeks	No training	TUGT, in-chair sitting-to-standing and squats, Berg scale

*A: Intervention Group; B: Control Group.*

*TUGT, group timed-up-and-go test; FTSST, five-times-sit-to-stand test; RMS, root mean square; AEMG, average electromyographic activity; IEMG, integrated electromyogram; CRP, C-reactive protein; TNFα, tumor necrosis factor; MFES, Modified Falls Efficacy Scale; PT, peak torque; AP, average power; TW, total work.*

**TABLE 2:** Characteristics of the included studies of Chinese herbal medicine and acupuncture.

Study	Age	Diagnostic criteria	Intervention		Outcome measures	Components/points
			Intervention group	Control group		
Chen, 2020	78 ± 5.21	AWGS	Bu-Zhong-Yi-Qi decoction combined with conventional treatment for 90 days	Conventional treatment: nutritional support and exercise for 90 days	Grip strength, 6-m gait speed	*Atractylodes macrocephala*, Astragalus, Radix pseudostellariae, *Ficus hirta*, Tortoise plastron, Degelatined deer-horn, Placenta Hominis, Angelica, Fructus Aurantii, Cimicifuga, Radix Bupleuri, Liquorice, Radix Moghaniae
Wen et al., 2018	73.11 ± 4.80	AWGS	Bu-Zhong-Yi-Qi decoction combined with conventional treatment for 12 weeks	Conventional treatment: nutritional support and exercise for 12 weeks	Grip strength, SPPB, Barthel	*Atractylodes macrocephala*, Astragalus, Radix pseudostellariae, *Ficus hirta*, Tortoise plastron, Degelatined deer-horn, Placenta Hominis, Angelica, Fructus Aurantii, Cimicifuga, Radix Bupleuri, Liquorice, Radix Moghaniae
Chen et al., 2021	66 ± 7.27	AWGS	Bu-Zhong-Yi-Qi decoction combined with conventional treatment for 90 days	Conventional treatment: nutritional support and exercise for 90 days	IL-6, TNF-α	*Atractylodes macrocephala*, Astragalus, Radix pseudostellariae, *Ficus hirta*, Tortoise plastron, Degelatined deer-horn, Placenta Hominis, Angelica, Fructus Aurantii, Cimicifuga, Radix Bupleuri, Liquorice, Radix Moghaniae
Ren et al., 2016	73.45 ± 3.46	AWGS	Bazhen decoction combined with conventional treatment for 12 weeks	Conventional treatment: nutritional support and exercise for 12 weeks	Grip strength, 6-m gait speed	*Atractylodes macrocephala*, Renshen, Liquorice, Poria cocos, Chuanxiong, Radix Rehmanniae Praeparata, Angelica, Radix Paeoniae Alba
Li, 2020	71.87 ± 5.26	Unspecified	Bazhen decoction combined with conventional treatment for 8 weeks	Conventional treatment: walk and Yi Jin Jing for 8 weeks	6-min walking distance, ADL, sit and reach	*Atractylodes macrocephala*, Renshen, Liquorice, Poria cocos, Chuanxiong, Radix Rehmanniae Praeparata, Angelica, Radix Paeoniae Alba
Liang et al., 2019	72.24 ± 3.20	AWGS	Bazhen decoction combined with conventional treatment for 12 weeks	Conventional treatment: nutritional support and exercise for 12 weeks	Grip strength, 6-m gait speed, ADL	*Atractylodes macrocephala*, Renshen, Liquorice, Poria cocos, Chuanxiong, Radix Rehmanniae Praeparata, Angelica, Radix Paeoniae Alba
Soares Mendes Damasceno et al., 2019	72 ± 7.90	EWGSOP	Acupuncture	No training	TUGT, Grip strength, IL6, IL10, and TNF-α	R3, BP3, BP6, VB34, F8, E36, TA6
Liu et al., 2020	68.12 ± 5.84	ISCCWG	Acupuncture combined with exercise (aerobic exercise, resistance exercise and balance training)	acupuncture combined with exercise (aerobic exercise, resistance exercise, and balance training)	Fugl-Meyer, Berg Scale, 4-m gait speed	Group A: ST36, K13, SP6**** Group B: ST31, ST32, GB34

A total of 20 trials were randomized controlled trials, and one study was a before–after study in the same patient. There were 20 randomized controlled studies mentioned randomization, but four of these studies (Ren et al., 2016; Wang et al., 2016; Zhao et al., 2016; Chen, 2020) did not provide detailed information. Only one study (Fang et al., 2020) provided a specific method of allocation concealment, this was not reported in most of the studies. All of the studies analyzed baseline information, and comparability of the baseline characteristics between the groups was an indicator of whether randomization was actually achieved. Imperfect diagnostic criteria led to uncertain additional risks in seven studies. Only one study (Soares Mendes Damasceno et al., 2019) specifically mentioned that the trial was single-blinded, but the lack of blinding was considered a relatively low risk factor for reducing the quality of the evidence. Overall, the low risk level accounted for about 53% of all risk levels assessed, and the uncertainty was about 46%, as shown in Table 3. All of the studies had small sample sizes, with the largest sample size being 214 and the smallest sample size being 15, and the smaller sample sizes reduced statistical efficacy and limited the reliability of the results. A total of 11 studies used multiple methods of combined treatment; the main confounding factors were not clearly described; and the results were not adjusted for confounding factors. Most of the included studies were conducted in China, so the effect of ethnicity may have added to the limitations of the results. A total of nine studies reported ethical approval (Gong et al., 2011; Zhu et al., 2016; Wen et al., 2018; Liang et al., 2019; Soares Mendes Damasceno et al., 2019; Fang et al., 2020; Liu et al., 2020; Chen et al., 2021; Morawin et al., 2021). According to the GRADE working group guidelines, the favorable factors of a larger sample size, clear and direct data, and high consistency of results improve the quality grade of grip strength results. The evidence for grip strength was of moderate quality among the results generated from these studies, while the evidence for the remaining results was of low quality, as shown in Tables 4–6.

**TABLE 3:** Risk of bias evaluation for the included randomized controlled trials (RCTs).

Projects	High risk	Low risk	Unclear risk
Random sequence generation	1	19	0
Allocation concealment	0	1	19
Blinding of participants and personnel	0	1	19
Blinding of outcome assessment	0	0	20
Incomplete outcome data	0	20	0
Selective reporting	0	20	0
Other bias	0	13	7
Total	1 (0.007%)	74 (53%)	65 (46%)

**TABLE 4:** Muscle strength for sarcopenia.

Outcomes	Illustrative comparative risks* (95% CI)	No. of participants (studies)	Quality of the evidence (GRADE)
	Corresponding risk (TCM)		
Grip strength	The mean grip strength in the intervention groups was 2.00 higher (0.44–3.55 higher)	641 (10 studies)	⊕⊕⊕⊝ moderate^a^
Squats	The mean squats in the intervention groups were 2.25 higher (1.12–3.39 higher)	209 (3 studies)	⊕⊕⊝⊝ low^a,b^
In-chair sitting-to-standing	The mean in-chair sitting-to-standing in the intervention groups was 2.45 higher (1.51–3.39 higher)	209 (3 studies)	⊕⊕⊝⊝ low^a,b^
Peak torque of knee extensor muscle group (60°/s)	The mean 60°/speak torque of knee extensor muscle group in the intervention groups was 10.12 higher (0.90–19.35 higher)	121 (2 studies)	⊕⊕⊝⊝ low^a,b^

*The basis for the assumed risk (e.g., the median control group risk across studies) is provided in footnotes. The corresponding risk (and its 95% CI) is based on the assumed risk in the comparison group and the relative effect of the intervention (and its 95% CI).

*CI, Confidence interval.*

*GRADE Working Group grades of evidence.*

*High quality: Further research is very unlikely to change our confidence in the estimate of effect.*

*Moderate quality: Further research is likely to have an important impact on our confidence in the estimate of effect and may change the estimate.*

*Low quality: Further research is very likely to have an important impact on our confidence in the estimate of effect and is likely to change the estimate.*

*Very low quality: We are very uncertain about the estimate.*

*^a^The included studies did not mention the allocation covert method or blind method, which made the bias risk of the study uncertain.*

*^b^Small sample size.*

**TABLE 5:** Physical performance for sarcopenia.

Outcomes	Illustrative comparative risks* (95% CI)	No. of participants (studies)	Quality of the evidence (GRADE)
	Corresponding risk (TCM)		
6-m walking speed	The mean 6-m walking speed in the intervention groups was 0.21 higher (0.2–0.22 higher)	416 (2 studies)	⊕⊕⊝⊝ Low^a,b^
TUGT	The mean TUGT in the intervention groups was 2.81 lower (4.08–1.55 lower)	69 (2 studies)	⊕⊕⊝⊝ Low^a,b^
Sit and reach	The mean sit and reach in the intervention groups was 1.39 higher (0.9–1.88 higher)	228 (3 studies)	⊕⊕⊝⊝ Low^a,b^

*The basis for the assumed risk (e.g., the median control group risk across studies) is provided in footnotes. The corresponding risk (and its 95% confidence interval) is based on the assumed risk in the comparison group and the relative effect of the intervention (and its 95% CI).

*CI, Confidence interval.*

*GRADE Working Group grades of evidence.*

*High quality: Further research is very unlikely to change our confidence in the estimate of effect.*

*Moderate quality: Further research is likely to have an important impact on our confidence in the estimate of effect and may change the estimate.*

*Low quality: Further research is very likely to have an important impact on our confidence in the estimate of effect and is likely to change the estimate.*

*Very low quality: We are very uncertain about the estimate.*

*^a^The included studies did not mention the allocation covert method or blind method, which made the bias risk of the study uncertain.*

*^b^Small sample size.*

**TABLE 6:** Muscle mass for sarcopenia.

Outcomes	Illustrative comparative risks* (95%CI)	No. of Participants (studies)	Quality of the evidence (GRADE)
	Corresponding risk (TCM)		
Muscle mass	The mean muscle mass in the intervention groups was 0.52 higher (0.1 lower to 1.14 higher)	472 (6 studies)	⊕⊕⊝⊝ low^a,b^

*The basis for the assumed risk (e.g., the median control group risk across studies) is provided in footnotes. The corresponding risk (and its 95% confidence interval) is based on the assumed risk in the comparison group and the relative effect of the intervention (and its 95% CI).

*CI, Confidence interval.*

*GRADE Working Group grades of evidence.*

*High quality: Further research is very unlikely to change our confidence in the estimate of effect.*

*Moderate quality: Further research is likely to have an important impact on our confidence in the estimate of effect and may change the estimate.*

*Low quality: Further research is very likely to have an important impact on our confidence in the estimate of effect and is likely to change the estimate.*

*Very low quality: We are very uncertain about the estimate.*

*^a^ The included studies did not mention the allocation covert method and blind method, which made the bias risk of the study uncertain.*

*^b^Three studies, Ren et al. (2016), Wen et al. (2018), and Chen (2020), showed an increase in muscle mass, while the fourth study Liang et al. (2019) showed no significant change in muscle mass.*

### Outcome Characteristics and Major Findings

There were six studies (Ren et al., 2016; Wen et al., 2018; Liang et al., 2019; Chen, 2020; Li, 2020; Chen et al., 2021) regarding Chinese herbal medicine for treating sarcopenia conducted on the basis of routine rehabilitation (exercise and nutrition), and three studies each used Ba Zhen decoction and Bu Zhong Yi Qi decoction. To investigate the effect of herbs on muscle strength and muscle mass, four studies (Ren et al., 2016; Wen et al., 2018; Liang et al., 2019; Chen, 2020) measured grip strength and the appendicular skeletal muscle mass index (ASMI kg/m^2^). Physical function was assessed in a variety of ways, with four studies (Ren et al., 2016; Liang et al., 2019; Chen, 2020; Li, 2020) using walking speed, three studies (Wen et al., 2018; Liang et al., 2019; Li, 2020) using activities of daily living (ADL), and one study (Wen et al., 2018) also using the short physical performance battery (SPPB). The results of five studies (Ren et al., 2016; Wen et al., 2018; Liang et al., 2019; Chen, 2020; Li, 2020) showed a significant increase in muscle strength and physical function in the herbal group compared with the control group (*p* < 0.05). Meanwhile, the results of three studies (Ren et al., 2016; Wen et al., 2018; Chen, 2020) showed a significant increase in muscle mass in the herbal group compared to the control group, while one study showed (Liang et al., 2019) no significant change in muscle mass with herbal medicine. The remaining study (Chen et al., 2021) investigated the effect of Bu Zhong Yi Qi decoction on inflammatory factors in patients with sarcopenia, and after taking it, the patients had lower serum IL-6 and TNF-α levels than the control group (*p* < 0.05).

There were 13 studies focused on Chinese traditional exercise (TCE). Among them, ten studies (Yan et al., 2007; Gong et al., 2011; Jing et al., 2011; Liu et al., 2016; Wang et al., 2016; Yang and Chen, 2016; Zhao et al., 2016; Zhu et al., 2017, 2018; Fang et al., 2020) involved Yi Jin Jing; two studies (Zhu et al., 2016; Morawin et al., 2021) involved Tai Chi; and one study (Zhou et al., 2020) involved Ba Duan Jing (BDJ). Among the 10 studies on the use of Yi Jin Jing for sarcopenia, eight studies used Yi Jin Jing only; one study (Zhao et al., 2016) involved a combination of Tuina, Yi Jin Jing, and resistance exercise; and another study (Yan et al., 2007) used Yi Jin Jing combined with Tuina. In two of the studies (Yang and Chen, 2016; Zhu et al., 2018), the control group underwent Tuina intervention. To evaluate physical function, four studies (Jing et al., 2011; Zhao et al., 2016; Zhu et al., 2016; Morawin et al., 2021) used a 6-m walking speed test; three studies (Zhu et al., 2016; Fang et al., 2020; Zhou et al., 2020) used the Time Up and Go Test (TUGT); and three studies (Jing et al., 2011; Wang et al., 2016; Li, 2020) used sit and reach. The scales used in these studies included the Berg Balance Scale, Modified Falls Efficacy Scale (MFES), Functional Gait Assessment (FGA), Barthel Index, Five Times Sit-to-Stand Test (FTSST), Tetrax index, and EPESE Physical Assessment Scale. Ten studies showed significant improvements in physical performance after the intervention compared with their respective control groups (*p* < 0.05). Yang and Chen (Yang and Chen, 2016) used walking steps to assess physical performance, but no time frame was given; lower extremity muscle strength scores were used to assess muscle strength, but no specific measurements or scoring methods were given. To evaluate muscle strength, six studies (Wang et al., 2016; Zhao et al., 2016; Zhu et al., 2016, 2017; Zhou et al., 2020; Morawin et al., 2021) used grip strength; four studies (Yan et al., 2007; Jing et al., 2011; Wang et al., 2016; Zhu et al., 2017) used the number of in-chair sitting-to-standing and squats in 15 s; and two studies (Gong et al., 2011; Liu et al., 2016) used the Biodex isometric muscle strength testing system. There were 10 studies showed that after exercise, patients’ muscle strength increased significantly compared with their respective control groups (*p* < 0.05). To evaluate muscle mass, two studies (Zhao et al., 2016; Morawin et al., 2021) used the ASMI (kg/m^2^), but their findings showed no significant change in muscle mass after the intervention compared to the control group. In the Tai Chi study, one of the studies showed no improvement in grip strength in the Tai Chi group compared with the control group after 8 weeks of exercise, but there was a significant improvement in iliopsoas muscle strength (*p* < 0.0001).

Among the included studies, two studies were on acupuncture. In one study, acupuncture combined with exercise (aerobic exercise, resistance exercise, and balance training) and two groups of acupuncture points were compared. The results showed that patients who received acupuncture at the ST36 showed better 4-m gait speed than those in the other group (Liu et al., 2020). Another clinical study, which did not choose acupuncture at ST36 for treating sarcopenia, showed that acupuncture did not increase muscle strength and physical function (Soares Mendes Damasceno et al., 2019).

## Discussion

**FIGURE 2:**
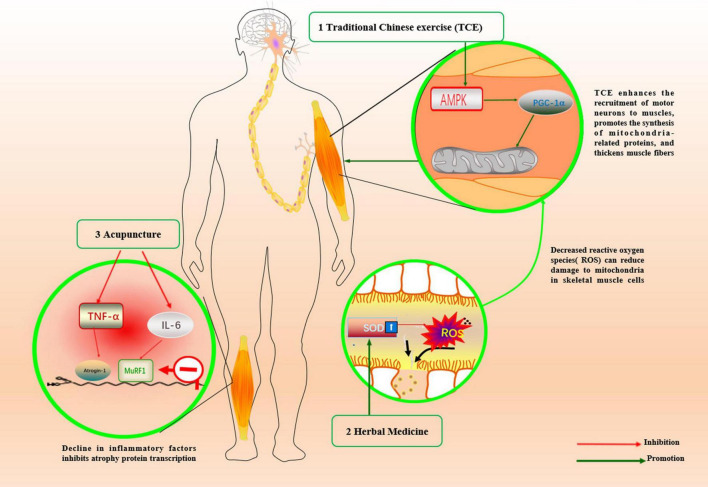
Mechanism of traditional Chinese medicine in the treatment of sarcopenia.

TCM may be used to delay sarcopenia by regulating the synthesis and degradation of muscle-related proteins, replenishing nutrients, promoting blood circulation, and eliminating inflammation, as shown in Figure 2.

Traditional Chinese exercises have originated from traditional Chinese medicine, and include Wu Qin Xi, Yi Jin Jing, Ba Duan Jin, and Tai Chi. These are gymnastic exercise consisting of various components such as endurance, resistance, balance, flexibility, breathing, and meditation (Nascimento et al., 2019). Exercise has a positive impact on the health of older adults, but the body’s motor organs and functions decline significantly with aging; as a result, it is important to emphasize the appropriate form and intensity of exercise for older adults (Colleluori and Villareal, 2021). Yi Jin Jing is a kind of static exercise. After high-intensity interval static exercise in aging rats, the PGC-1α/FNDC5/UCP1 signaling pathway was activated, PGC-1α was upregulated, mitochondria increased, muscle fiber thickening was observed, and the skeletal muscle atrophy state was improved (Liu et al., 2021). The results of the included studies consistently show that Yi Jin Jing is an effective way to build muscle strength to prevent muscle atrophy, but there are limitations to the findings. Yi Jin Jing may be more helpful in improving lower limb strength and less effective in improving upper limb strength, considering that after practicing Yi Jin Jing, grip strength did not improve significantly, but the number of squats and in-chair sitting-to-standing within 15-s period increased significantly (Jing et al., 2011). Zhao et al. (2016) used a complex intervention that combined Tuina with Yi Jin Jing as one intervention and then combined it with another intervention, resistance exercise. The results showed that the three treatment measures group was more effective than Tuina combined with Yi Jin Jing or resistance exercise in improving muscle strength and physical function (*p* < 0.05). Whether the beneficial effects of the combination of two or more exercises on the body are caused by the own advantages of the different exercises or simply by the increase in the total amount of exercise cannot be determined. Moreover, the use of multiple treatments simultaneously can easily lead to poor patient compliance and reduce the credibility of the study results. In this study, only walking speed increased, and was statistically significant in the Tuina combined with Yi Jin Jing group compared to the no intervention group; however, there was a significant difference in grip strength compared to pre-intervention. There was an increase in muscle strength after using Tuina combined with Yi Jin Jing intervention, as evidenced by an increase in the number of chair stand tests performed over a 15-s period compared with the pre-treatment period (Yan et al., 2007). However, this evidence does not allow for precise conclusions that Yi Jin Jing combined with Tuina can improve muscle strength, despite significant differences in indicators before and after the intervention. Besides the lack of favorable evidence from the control group, there were other confounding factors such as the difference in the severity of the disease before and after. Yi Jin Jing was more effective than Tuina in FGA scores and total scores (Zhu et al., 2018). However, the degrees of increase in walking times and mean lower limb strength score in the massage group were significantly higher than that in the Yinjjing group (*p* < 0.05) in another study (Yang and Chen, 2016). Therefore, additional evidence is needed to determine whether Yi Jin Jing or Tuina is more effective.

In studies of Tai Chi for balance problems in the elderly, the diversity of its style may lead to uncertainties in its therapeutic effects. Morawin et al. (2021) used Yang-style 24-form Tai Chi after which patients had increased walking speed and decreased fat mass. Another study (Zhu et al., 2016) used a simple 8-style Taijiquan and showed significant improvements in lower extremity muscle strength and physical performance in the TC group compared to the control group, but the gender of their included population was all male making the results limited. Yang-style 24-form Tai Chi is the form most commonly used to improve balance in the elderly (Liu and Frank, 2010; Sherrington et al., 2019). However, many studies currently use simplified Tai Chi for treatment. A meta-analysis showed that traditional Tai Chi was more effective than simplified Tai Chi in preventing falls in the elderly (Li et al., 2021). However, some studies have also concluded that customized therapeutic Tai Chi is more effective than traditional Tai Chi in reducing the incidence of falls in older adults who are at a higher risk of falling (Li et al., 2018; Penn et al., 2019). During Tai Chi practice, the stability of individual gait is lower than that of normal gait (Yang and Liu, 2020). Although the low stability of gait in Tai Chi increases the risk of falls, it stimulates the body’s postural control system (Jahnke et al., 2010). To maintain balance, the body must autonomously adapt to make postural adjustments and enhance neuromuscular control so as to improve the ability to control body balance. A plantar pressure test system of the elderly after practicing Tai Chi showed that their heel impulse was greater than that of the no-intervention group, suggesting that it promoted better neuromuscular control (Li et al., 2016). This also reflects the yin and yang theory of Tai Chi, which seeks balance in the body between stability and instability. A different study demonstrated that 48 weeks of Taijiquan stability training significantly improved the stability limits of older adults, with 32, 68, and 19% increases in endpoint excursion, movement velocity, and directional control, respectively, compared with the baseline (Li, 2014).

We found only one study (Zhou et al., 2020) on the use of BDJ for sarcopenia. It may be due to the fact that BDJ does not have the same strong influence and wide dissemination as Taijiquan and Yi Jin Jing. The results from a 12-week clinical trial of sitting BDJ showed that it improved balance but not lower limb strength, possibly because sitting BDJ focused more on coordination than on muscle strengthening (Bao et al., 2020). Elderly individuals who have difficulty moving are advised to practice sitting BDJ.

According to Chinese medicine theory, Ba Zhen decoction and Bu Zhong Yi Qi decoction are commonly used Chinese herbal formulas to benefit Qi energy and strengthen the function of spleen and stomach to reduce weakness. The results of the included studies can only indicate that herbal medicine has a beneficial effect on improving muscle strength and physical function in patients based on exercise and nutritional supplementation. Bu Zhong Yi Qi decoction was able to reduce inflammatory factors in patients (Chen et al., 2021), but due to the small sample size, there was a risk of deviation. Animal studies have shown that Astragalus, which is contained in Bu Zhong Yi Qi decoction, has antioxidant and anti-aging effects. Astragalus polysaccharides, the main component of Astragalus, have been shown to significantly increase the activities of catalase (CAT), superoxide dismutase (SOD), and glutathione peroxidase (GPx), as well as anti-hydroxyl radicals in D-gal-induced aging mice (Li et al., 2012). Both Ba Zhen decoction and Bu Zhong Yi Qi decoction contain *Atractylodes macrocephala* and Chuanxiong. *Atractylodes macrocephala* can increase SOD activity, scavenge reactive oxygen radicals, and reduce malondialdehyde content in the erythrocytes of mice over 12 months of age (Li et al., 1996). In aging rats, the SOD content in the serum and rectus femoris muscle was increased by gavage of an herbal cuisine containing *Atractylodes macrocephala* and Astragalus, thus reducing oxidative damage and delaying the aging of skeletal muscles (Li and Liu, 2018). Chuangxiong contains tetramethylpyrazine (TMP), which can improve age-related musculoskeletal disorders in humans and prolong the lifespan (Liu et al., 2019).

The inability of acupuncture to relieve symptoms in patients with sarcopenia may be due to the selection of acupuncture points, but acupuncture may help regulate inflammatory cytokines IL-6 and TNF-α in their bodies (Soares Mendes Damasceno et al., 2019). This is because each point has a clear therapeutic effect, but it is important to combine multiple related points together for systematic acupuncture treatment depending on the condition of the disease. Liu et al. (2020) compared two different groups of acupuncture points and showed that the group with acupuncture containing the Zusanli Point (ST36) outperformed the other group in terms of walking speed. ST36 is the most frequently used acupoint in skeletal muscle-related diseases and is often used to increase muscle function and strength (Ahmedov, 2010). This may be because ST36 is located on the lateral side of the lower leg, the superficial layer is distributed with the lateral sural cutaneous nerve, and the deep layer has branches of the anterior tibial artery and vein. Acupuncture at this point can stimulate the nerve to activate the motor cortex, stimulate muscle contraction, and increase the blood flow of skeletal muscles to improve muscle strength and muscle mass of the lower limb (Noguchi et al., 1999; Ohkubo et al., 2009; Sun et al., 2019). There is a lack of clinical cases of acupuncture for sarcopenia, but there is evidence from relevant animal experiments. These studies are mostly animal models of skeletal muscle atrophy caused by other diseases, which is still different from natural aging-induced skeletal muscle atrophy. Although there are some limitations, the mechanism of sarcopenia treatment by acupuncture can be elucidated to some extent. Muscle-specific E3 ubiquitin ligases, such as atrogin-1 and MuRF1, play a key role in muscle atrophy (Egerman and Glass, 2014). Electroacupuncture in the ST36 and SP9 of rats with diabetes-induced muscular atrophy has been shown to reduce the expression of MURF-1, prevent the degradation of the myosin heavy chain, and delay the process of muscular atrophy (Chen et al., 2019). Autophagy can remove damaged proteins and provide materials for protein self-renewal to inhibit apoptosis of skeletal muscle cells. It has also been reported that electroacupuncture at ST36 and GB30 points can promote autophagy to improve gastrocnemius atrophy in rats with dystrophic muscular atrophy (Zhao et al., 2015).

### Limitations and Directions for Future Research

This is the first systematic review of TCM for the treatment of sarcopenia. This review included clinical and preclinical studies of three major types of TCM approaches for sarcopenia—traditional Chinese Qigong exercises, acupuncture, and Chinese herbal medicine—using a reproducible search method in the available databases. Here, we highlighted the role and problems of TCM in the field of sarcopenia by summarizing and analyzing the current literature. In addition, we used the current evidence to explain the potential mechanisms. However, the available preclinical study models are not fully consistent with the pathogenesis of sarcopenia and there are mechanistic differences. The inclusion criteria for our study were strict, and we excluded studies on TCM for the treatment of secondary sarcopenia (such as metabolic disorders and cancer), thus neglecting the use of TCM for sarcopenia in these diseases. Although global diagnostic criteria for sarcopenia are now available, some earlier studies relied on expert scholarly views of sarcopenia at the time, which has led to an increased risk of uncertain bias. The limited number of eligible studies, the small amount of data, the intervention methods, and the high heterogeneity of the measurement methods prevented a meta-analysis of the existing literature. To improve the quality of evidence for future studies, there is a need for consensus on inclusion criteria for patients with sarcopenia and standardization of muscle strength, muscle function, and muscle mass testing. In the future, a large number of high-quality studies will make it possible to conduct meta-analyses of different methods such as traditional Chinese Qigong exercises, herbal medicine, and acupuncture separately, providing a higher level of evidence to determine whether TCM is effective in treating sarcopenia. Further comparative studies between several methods could be added for superiority analysis.

## Conclusion

Traditional Chinese Qigong exercises and Chinese herbal medicine have a positive effect on physical performance and muscle strength in older adults with sarcopenia. Future high-quality multicenter RCTs with large samples are needed to determinate whether acupuncture and other therapies are effective in treating sarcopenia.

## Data Availability Statement

The original contributions presented in the study are included in the article/supplementary material, further inquiries can be directed to the corresponding author.

## Author Contributions

LF conceived the review. SL, ZL, YM, and RZ searched the literature. CG was responsible for data analysis and writing of the first draft. XX, CG, and YM performed the data management and figure modification. LF and YM modified the manuscript. All authors contributed to the article and approved the submitted version.

## Conflict of Interest

The authors declare that the research was conducted in the absence of any commercial or financial relationships that could be construed as a potential conflict of interest.

## Publisher’s Note

All claims expressed in this article are solely those of the authors and do not necessarily represent those of their affiliated organizations, or those of the publisher, the editors and the reviewers. Any product that may be evaluated in this article, or claim that may be made by its manufacturer, is not guaranteed or endorsed by the publisher.
